# Bridging genetic knowledge gaps in a biodiversity hotspot through conservation training

**DOI:** 10.1093/biosci/biag035

**Published:** 2026-04-13

**Authors:** Alba Enguídanos García, Víctor Cuesta-Porta, Jeancarlos Abrego L., Milton Almanza, Kensy Zuleny Alvelo Villegas, Joan Antaneda H., Leighanne Bonner, Odelkys J Carpintero, Gustavo Nelson Collado, Darío Córdoba González, Maira Díaz Vergara, Zedna I Guerra-Lima, Juan de Dios Noriega, Daniela Sofía de León Herrera, Alana I Domingo, Ovidio Durán, Anette Garrido-Trujillo, Yorlenis González, Sara Ceciel Justo Riverol, Analía Guerrero Romero, Noemí León Correoso, Laura E P Molina, Edilma Montalvo, Claudia M Pérez-González, Marleny Rivera, Javier Leonardo Vásquez Acosta, Mario Vásquez, Ana Vega-Atencio, Wendy Zhuo, Carles Galià-Camps

**Affiliations:** Departament de Biologia Evolutiva, Ecologia i Ciències Ambientals, Facultat de Biologia, Universitat de Barcelona (UB), Avinguda Diagonal 643, 08028 Barcelona, Spain; Institut de Recerca de la Biodiversitat (IRBio), Universitat de Barcelona, Barcelona, Spain; Departament de Biologia Evolutiva, Ecologia i Ciències Ambientals, Facultat de Biologia, Universitat de Barcelona (UB), Avinguda Diagonal 643, 08028 Barcelona, Spain; Institut de Recerca de la Biodiversitat (IRBio), Universitat de Barcelona, Barcelona, Spain; Departamento de Zoología, Facultad de Ciencias Naturales, Exactas y Tecnología, Universidad de Panamá (UP), Panamá, República de Panamá; Departamento de Biotecnología, Facultad de Ciencias de la Salud, Universidad Latina de Panamá (ULat), Panamá, República de Panamá; Facultad de Ciencias Naturales, Exactas y Tecnología, Universidad de Panamá, Panamá, República de Panamá; Comisión Panamá – Estados Unidos para la Erradicación y Prevención del Gusano Barrenador del Ganado (COPEG), Panamá, República de Panamá; Departamento de Biotecnología, Facultad de Ciencias de la Salud, Universidad Latina de Panamá (ULat), Panamá, República de Panamá; Facultad de Ciencias Naturales, Exactas y Tecnología, Universidad de Panamá, Panamá, República de Panamá; Autoridad de los Recursos Acuáticos de Panamá (ARAP), Panamá, República de Panamá; Departamento de Zoología, Facultad de Ciencias Naturales, Exactas y Tecnología, Universidad de Panamá (UP), Panamá, República de Panamá; Museo de Malacología de la Universidad de Panamá (MUMAUP), Panamá, República de Panamá; Facultad de Biociencias y Salud Pública, Universidad Especializada de las Américas, Panamá, República de Panamá; Autoridad de los Recursos Acuáticos de Panamá (ARAP), Panamá, República de Panamá; Departamento de Biotecnología, Facultad de Ciencias de la Salud, Universidad Latina de Panamá (ULat), Panamá, República de Panamá; Departamento de Biotecnología, Facultad de Ciencias de la Salud, Universidad Latina de Panamá (ULat), Panamá, República de Panamá; Departamento de Genética y Biología Molecular, Facultad de Ciencias Naturales, Exactas y Tecnología, Universidad de Panamá, Panamá, República de Panamá; Departamento de Genética y Biología Molecular, Facultad de Ciencias Naturales, Exactas y Tecnología, Universidad de Panamá, Panamá, República de Panamá; Departamento de Genética y Biología Molecular, Facultad de Ciencias Naturales, Exactas y Tecnología, Universidad de Panamá, Panamá, República de Panamá; Museo de Invertebrados G. B. Fairchild, Universidad de Panamá, Panamá, República de Panamá; Smithsonian Tropical Research Institute, Balboa, Ancon, Panamá, República de Panamá; Estación Científica Coiba AIP, Panamá, República de Panamá; Facultad de Ciencias del Mar, Universidad Marítima Internacional de Panamá (UMIP), Panamá, República de Panamá; Facultad de Ciencias Naturales, Exactas y Tecnología, Universidad de Panamá, Panamá, República de Panamá; Centro de Investigación de Criobiología (CIC), Facultad de Ciencias Naturales, Exactas y Tecnología, Universidad de Panamá, Panamá, República de Panamá; Autoridad de los Recursos Acuáticos de Panamá (ARAP), Panamá, República de Panamá; Facultad de Ciencias Naturales, Exactas y Tecnología, Universidad de Panamá, Panamá, República de Panamá; Departamento de Genética y Biología Molecular, Facultad de Ciencias Naturales, Exactas y Tecnología, Universidad de Panamá, Panamá, República de Panamá; Centro de Investigación de Criobiología (CIC), Facultad de Ciencias Naturales, Exactas y Tecnología, Universidad de Panamá, Panamá, República de Panamá; Smithsonian Tropical Research Institute, Balboa, Ancon, Panamá, República de Panamá; Programa de Biología Marina, Facultad de Ciencias Naturales e Ingeniería, Universidad Jorge Tadeo Lozano (UJTL), Cr. 2 #11-68, Santa Marta, Colombia; Facultad de Ciencias Naturales, Exactas y Tecnología, Universidad de Panamá, Panamá, República de Panamá; Smithsonian Tropical Research Institute, Balboa, Ancon, Panamá, República de Panamá; Departamento de Biotecnología, Facultad de Ciencias de la Salud, Universidad Latina de Panamá (ULat), Panamá, República de Panamá; Departamento de Biodiversidad y Biología Evolutiva, Museo Nacional de Ciencias Naturales (MNCN-CSIC), Calle José Gutiérrez Abascal, 228006 Madrid, Spain; Centre d'Estudis Avançats de Blanes (CEAB-CSIC), Accés Cala St. Francesc 14, 17300 Blanes, Spain

**Keywords:** DNA barcoding, conservation genetics, education initiatives, invertebrate biodiversity, invasive species

## Abstract

The Mesoamerican biodiversity hotspot is extraordinarily rich, yet most invertebrate genetic diversity remains invisible, hampering effective conservation planning amid accelerating biodiversity loss. How can this hidden diversity be revealed while simultaneously building local scientific capacity? *Panama BioResearch*, a hands-on molecular course, addressed this issue by embedding DNA barcoding within training and conservation contexts. Participants collected terrestrial and marine invertebrates across three protected areas and generated 158 DNA barcode sequences, two-thirds of which represented first genetic records for their species. Comparisons with public databases revealed striking under-representation of Mesoamerican taxa, especially non-iconic groups with key ecosystem roles. Barcoding also enabled the rapid detection of two invasive species, prompting immediate management responses. Beyond documenting biodiversity, this experience demonstrates that small, low-cost educational initiatives can produce actionable data, foster local expertise, and inform conservation priorities. Embedding molecular tools in education provides a scalable model for linking research, training, and management in species-rich but data-deficient regions.

## Decoding Panama’s Genetic Gap

Panama lies at the heart of one of the most biologically rich regions on Earth: the Mesoamerican biodiversity hotspot (Myers et al. 2000). Despite its extraordinary richness, much of Panama’s biodiversity remains poorly characterized, particularly at the genetic level (Murphy et al. 2016). This gap is most evident among invertebrates, organisms that underpin ecosystem functioning through processes such as decomposition, nutrient cycling, pollination, and soil formation (Lavelle et al. 2006, Struijk et al. 2024). However, they often stay away from the conservation focus and remain largely invisible in management strategies (Jarić et al. 2018).

Genetic data are increasingly essential for effective conservation (Hebert et al. 2003, Jaffé et al. 2019, Shaw et al. 2025). DNA-based approaches can reveal hidden diversity, identify evolutionarily distinct populations, and detect biological invasions at early stages (Hajibabaei et al. 2007, Silva-Brandão et al. 2013, Weigand et al. 2019). However, access to molecular infrastructure, training, and bioinformatic tools remains uneven across the globe. In Panama and across much of the tropics, limited access to molecular training has constrained local participation in conservation genetics, reinforcing geographic biases in global biodiversity databases. Education-centered molecular initiatives offer a promising solution (Henter et al. 2016). By coupling hands-on training with real biodiversity research, such initiatives can generate high-quality genetic data while simultaneously building local capacity (Erasmus 2021, Wright et al. 2024).

Our education-centered DNA barcoding initiative, *Panama BioResearch*, brought molecular tools directly into the hands of locals. Through this course, participants generated genetic data from common invertebrates across multiple habitats, uncovered undocumented genetic diversity, and detected invasive species with immediate management relevance. Our experience illustrates how small, accessible training programs can simultaneously build local capacity while producing relevant biodiversity data.

## Integrating education and conservation genetics

DNA barcoding has become a widely used tool for documenting biodiversity because it is simple, robust, and broadly applicable across taxonomic groups (Hebert et al. 2003). Using a short standardized DNA fragment (the mitochondrial cytochrome oxidase I (*cox1*) in animals), organisms that are difficult or impossible to identify morphologically can be linked to reference sequences in global databases, enabling rapid species identification, detection of cryptic diversity, and recognition of invasive taxa even in taxonomically challenging groups (Hajibabaei et al. 2007, Silva-Brandão et al. 2013, Weigand et al. 2019). Importantly, for students and early-career practitioners, the appeal of barcoding lies not only in its power but also in its accessibility. With a small number of laboratory steps, participants can move from a field-collected specimen to a searchable genetic sequence within days. By comparing newly generated sequences to global repositories, students engage directly with real biodiversity data, fostering interdisciplinary learning across ecology, genetics, and informatics while contributing meaningfully to publicly accessible resources (Chodkowski et al. 2022).

*Panama BioResearch* was designed with a clear educational purpose and aimed to remove any economic and technical barrier to molecular training while embedding real biodiversity research at every stage of instruction. Registration fees were kept low ($60), and full-tuition scholarships were offered to undergraduate students. As a result, the course brought together a diverse group of participants, including undergraduate students, university professors, and government environmental managers from Panama and neighboring countries.

**Figure 1:**
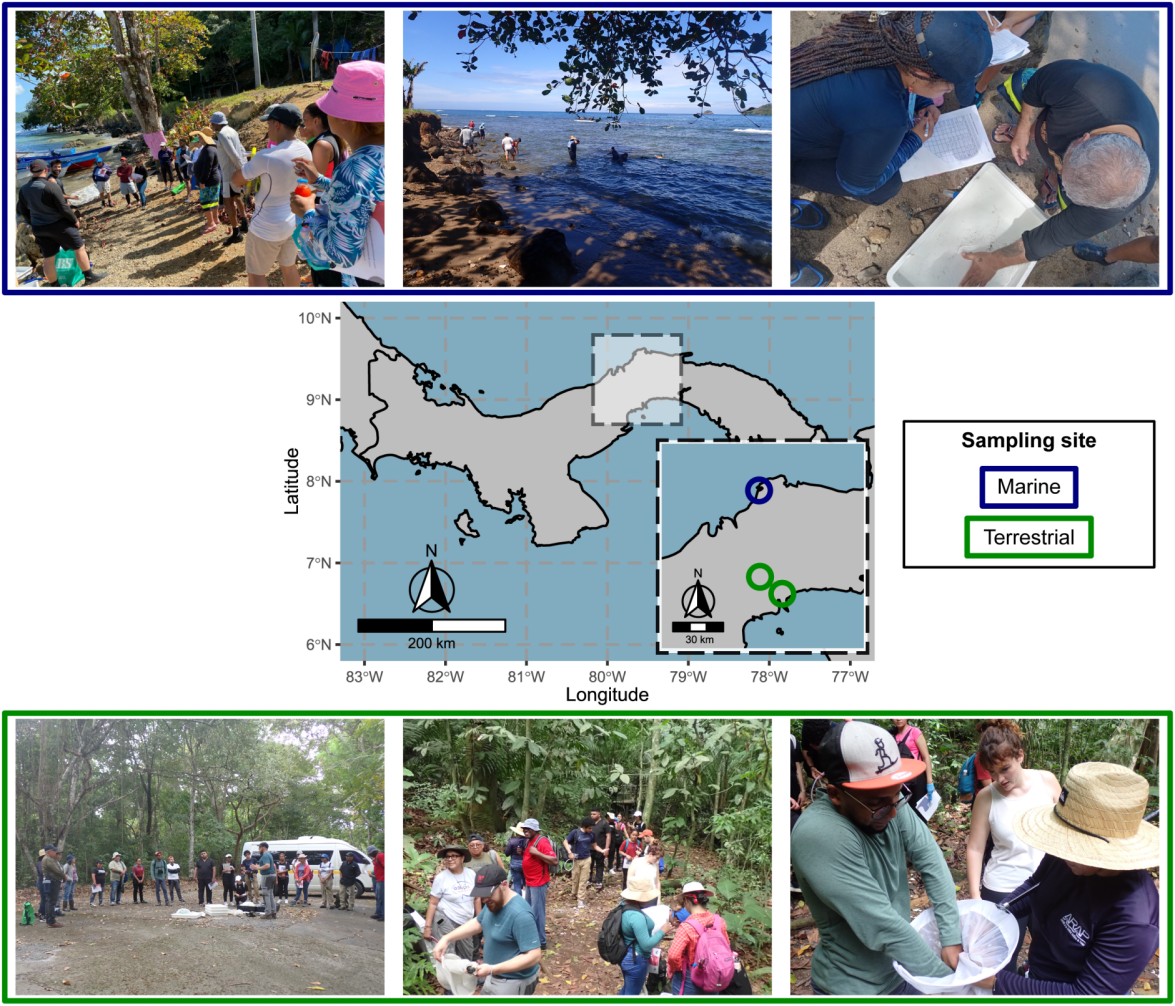
*Panama BioResearch* sampling sites and field activities. Photographs illustrate key moments during the course: the initial briefing, participants collecting specimens in the field, and participants annotating the samples’ metadata.

Field sampling was conducted in three protected areas representing both terrestrial and marine ecosystems: *Parque Natural Metropolitano, Parque Nacional Soberanía*, and the Caribbean coast near Portobelo (figure 1). Participants collected common invertebrates using low-impact methods, emphasizing biodiversity documentation while minimizing habitat disturbance. For many attendees, this was their first experience collecting specimens, extracting DNA, or performing basic bioinformatics. Importantly, all sequences generated during the course were deposited in the open-access repository NCBI, and protocols and teaching materials were shared freely among participants (especially professors) so they could incorporate them into their lectures. This openness was intentional. Rather than treating training and research as separate activities, *Panama BioResearch* sought to create a feedback loop in which education generates data, and data reinforce the value of education while also recognizing that their work contributed directly to global biodiversity resources. Globally, large barcoding initiatives, such as the International Barcode of Life (iBOL) (Adamowicz 2015), have made remarkable progress in building reference libraries, but these efforts remain heavily skewed toward temperate regions. By embedding DNA barcoding within education programs in biodiversity hotspots, small-scale courses like *Panama BioResearch* offer a practical way to help rebalance this inequity while fostering long-term local scientific independence in conservation genetics.

## Revealing hidden genetic diversity

Over the course of the *Panama BioResearch* training, participants collected hundreds of specimens of terrestrial and marine invertebrates. In just a few days, participants generated over a 150 DNA barcodes and deposited them in public databases. Even this modest sampling revealed a surprising breadth of invertebrate diversity, spanning cnidarians, molluscs, and multiple arthropod lineages.

**Figure 2:**
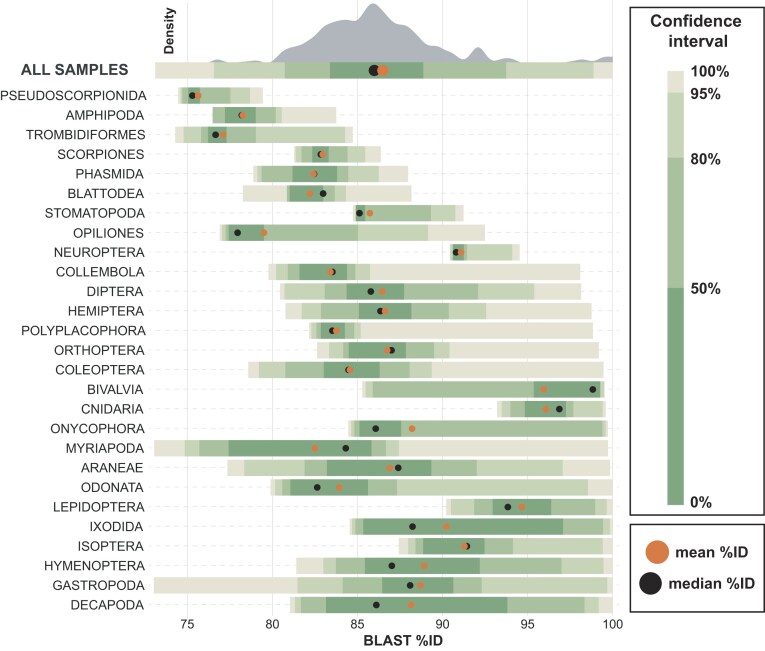
Genetic distinctiveness of the invertebrate samples collected during the course. For each taxonomic group, the plot shows how closely our sequences matched entries in public databases. We highlight the mean and median %ID, as well as the 100%, 95%, 80%, and 50% confidence intervals. Across all groups, most sequences lacked close matches, highlighting the large knowledge gap of Panama’s invertebrate genetic diversity.

When participants compared their sequences with existing reference libraries, a clear, striking pattern emerged. Most barcodes showed relatively low similarity to any existing database entry, suggesting that close genetic matches are uncommon and, therefore, there is a huge information gap regarding tropical genetic biodiversity. This pattern appeared again and again across major invertebrate groups (cnidarians, molluscs, and arthropods alike) rather than being driven by a single poorly sampled lineage. However, non-iconic taxa took the “misinformation prize” (figure 2). In practical terms, many of the organisms collected during the course had never been genetically documented before. Nearly two-thirds of the sequences represented the first genetic records for their species, whereas only seven sequences corresponded to previously documented haplotypes, underscoring the uniqueness of Panamanian genetic diversity.

What was most striking was that these discoveries came from common and easily collected organisms! If such levels of undocumented genetic diversity were present among commonly found invertebrates, much deeper layers of hidden genetic diversity almost certainly remain among smaller, rarer, and taxonomically neglected groups. Our observations reinforce that Panama and the broader Mesoamerican region are not only hotspots of species richness, but also reservoirs of unique evolutionary lineages that remain largely invisible at the genetic level and deserve targeted conservation attention (Myers et al. 2000, Hoban et al. 2022). The data generated through *Panama BioResearch*, a low-cost, standardized training-based framework, are a readily scalable approach that could be replicated through similar courses worldwide to accelerate the discovery of hidden genetic diversity in other understudied biodiversity hotspots.

## Early detection of invasive species

Beyond uncovering hidden native diversity, the course revealed unexpected specimens. Two invasive species were detected, with immediate management implications for a country willing to preserve its autochthonous biota.

The first was the terrestrial snail *Ovachlamys fulgens*, native to Japan. This species has spread globally through the horticultural trade and is known to damage crops (Rosa et al. 2022), and act as a vector for human pathogens (Kim et al. 2014). For participants, spotting this species in Parque Natural Metropolitano was a surprise, as it was the first confirmed record of *O. fulgens* in Panama, highlighting the need for increased monitoring. Notably, the director of the Parque Natural Metropolitano was among the barcoding course attendees, enabling this discovery to be reported and acted on immediately, highlighting how hands-on training of participants with a wide amalgam of professional backgrounds can facilitate management decisions.

The second case involved a marine bivalve belonging to the genus *Saccostrea*, detected on the Caribbean coast near Portobelo. These oysters are notorious fouling organisms capable of long-distance dispersal by ship hulls, as also reported for other sessile invasive species (Galià-Camps et al. 2024). Finding them close to the Panama Canal raises concerns about human-mediated species movement between oceans across the canal, reminding us that even well-studied waterways are vulnerable to hidden invasions (Riley et al. 2022).

These examples illustrate the practical power of DNA barcoding within an education-focused course. By combining fieldwork with molecular analysis, participants not only learned valuable skills but also generated actionable data that could inform immediate conservation and monitoring efforts even when morphological expertise was lacking. In this way, a short training course became a tool for both discovery and management.

## Implications for conservation and policy

*Panama BioResearch’s* findings carry several important implications for biodiversity science and conservation practice:

1. These observations suggest that invertebrate genetic diversity in the Mesoamerican hotspot is far richer, more geographically structured, and more evolutionarily distinct than is currently represented in global reference databases. The high proportion of novel barcode records and previously undocumented haplotypes recovered from common species highlights how tropical invertebrate diversity remains largely invisible at the genetic level. Conservation strategies that fail to incorporate this hidden diversity risk overlook evolutionarily significant units, underestimating regional endemism, and misallocating limited conservation resources.
2. The rapid detection of invasive species through DNA barcoding underscores the value of routine molecular monitoring, particularly in regions experiencing intense human-mediated species movement, such as Panama, where global trade, tourism, and the Panama Canal facilitate biological introductions. In just a few days, participants generated data that enabled park managers to respond to new introductions, a process that would have taken much longer using traditional morphological surveys.
3. The study highlights the effectiveness of integrating molecular training directly into conservation practice. Education-driven conservation genetics provides a scalable and cost-effective pathway to generate actionable biodiversity data while simultaneously building local technical capacity. By empowering local students, scientists, and environmental managers with accessible genetic tools, countries like Panama can take a leading role in documenting and protecting their own biodiversity. This approach promotes scientific sovereignty, reduces dependence on external research initiatives, and contributes high-value data to global biodiversity databases, helping to correct long-standing geographic biases in molecular biodiversity knowledge.

## Data Availability

The molecular data have been deposited into the public database GenBank.
